# Noise Radar Technology: Waveforms Design and Field Trials

**DOI:** 10.3390/s21093216

**Published:** 2021-05-06

**Authors:** Gaspare Galati, Gabriele Pavan, Kubilay Savci, Christoph Wasserzier

**Affiliations:** 1 Department of Electronic Engineering, Tor Vergata University and CNIT-Consortium for Telecommunications, Research Unit of Tor Vergata University of Rome, 00133 Rome, Italy; gaspare.galati@uniroma2.it; 2 Turkish Naval Research Center Command, Koc University, Istanbul 34450, Turkey; ksavci@ieee.org; 3 Fraunhofer Institute for High Frequency Physics and Radar Techniques FHR, 53343 Wachtberg, Germany; christoph.wasserzier@fhr.fraunhofer.de

**Keywords:** noise radar technology, waveforms design, autocorrelation function, leakage effect, peak-to-average power ratio, peak sidelobes level, continuous emission

## Abstract

Performance of continuous emission noise radar systems are affected by the sidelobes of the output of the matched filter, with significant effects on detection and dynamic range. Hence, the sidelobe level has to be controlled by a careful design of the transmitted waveform and of the transmit/receive parts of the radar. In this context, the average transmitted power has to be optimized by choosing waveforms with a peak-to-average power ratio as close to the unity as possible. However, after coherent demodulation and acquisition of the received signal and of the reference signal at the transmitting antenna port, the goodness (low sidelobes) of the output from the matched filter can be considerably reduced by the deleterious effects due to the radar hardware, including the analog-to-digital converter (ADC). This paper aims to solve the above problems from both the theoretical and the practical viewpoint and recommends the use of tailored waveforms for mitigating the dynamic range issues. The new findings are corroborated by the results from two noise radar demonstrators operating in Germany (rural environment) and in Turkey (coast and sea environment) and the related lessons learnt.

## 1. Introduction

An overview on noise radar technology (NRT) is summarized in the companion paper “Introduction to noise radar and its waveforms” [1] of this Special Issue, where main features of an NRT, advantages and limitations with respect the conventional radar systems and design problems are widely illustrated. The present paper describes the experimental field tests and the related results obtained transmitting the types of waveforms as defined in [1]. The trials were carried out in summer/autumn/winter 2020 at the Fraunhofer Institute for High Frequency Physics and Radar Techniques (FHR) in Wachtberg (Germany) and at the Turkish Naval Research Center Command (TNRCC) in Istanbul (Turkey) using two different noise radar demonstrators that will be described later. These demonstrators have been developed after consideration of the main requirements for modern radars, including multiple-input multiple-output (MIMO) radar [2], low probability of intercept/exploitation (LPI/LPE) radar [3] and noise radar (NR) [1]. The features include:

(a) waveform with good auto and cross correlation function (ACF/CCF), i.e., low peak sidelobes level (PSL) of the ACF to avoid that strong targets mask the weak ones, and good orthogonality (low CCF) to reduce mutual interference;
(b) radar resilience to electronic support measures (ESM) and to electronic counter measures (ECM) [4].

Regarding point (a), a possible solution uses “tailored” pseudorandom waveforms, with sidelobes suppression of the autocorrelation in a particular range interval. Tailored waveforms can be obtained by cyclic algorithms (CA), as described in [5], with the drawback of an uncontrolled use of the electromagnetic spectrum, causing a conflict with the band-occupancy constraints, often associated to each operating environment [6]. In [7], a different approach, named Band Limited Algorithm for Sidelobes Attenuation (BLASA), has been presented to mitigate the previous limitation. In [1], a much simpler technique to suppress the sidelobes of the ACF in a range interval of interest, named FMeth, has been proposed and will be considered in this work. Its main advantage over CA and BLASA is a low computational burden, especially for high time-bandwidth (BT) products.

The use of noise waveforms allows the radar designer to satisfy requirement (a). Nevertheless, there are some drawbacks to highlight. The most significant one is the limited exploitation of the power transmitter. The average transmitted power in a NR may be some 10 dB lower than in a FM-CW or pulse compression radar using the same power amplifier; this is due to the need to maintain most of the dynamic range of the transmitted noise waveform. When the system requirements include the best exploitation of the power, the peak power of each radiated waveform has to be limited with respect to the average power.

Let us denote as g(t) the noise waveform, defined by its samples g[k] with k=1, 2,…,N (being $N=F_{s}\cdot T$ and $F_{s}$ the sampling frequency). The peak-to-average power Ratio, or PAPR:

(1) $$PAPR=\frac{\underset{k}{\max}{\left|g\left[k\right]\right|}^{2}}{\frac{1}{N}\sum_{k=1}^{N}{\left|g\left[k\right]\right|}^{2}}\;$$

has to be set close to the unity to best exploit the power transmitter, i.e., to reduce the loss in signal-to-noise ratio (SNR). Of course, in the limit-case of phase-modulated noise waveforms (constant amplitude) [8,9], the PAPR is equal to the unity and there is no SNR loss.

Regarding the requirement (b), for a given bandwidth B, setting the desired ACF with low PSL and the low CCF, the randomness, inserted into the amplitude and the phase of the signal, enhances the low probability of intercept (LPI) and of exploitation (LPE) of the waveform making more and more difficult the identification and the spoofing of the radar signal. However, quantifying the LPI/LPE-goodness of a noise radar is a difficult task, beyond the scope of this paper, and a potential, general tool to this aim can be supplied by the information theory, i.e., using the marginal and joint entropy and the mutual information [10,11].

In NRT, the transmitting section of the system includes a digital waveform generator (core of the NRT) able to generate the In-phase (I) and Quadrature (Q) components of a signal having the requested values of bandwidth (B) and duration (T). This *reference signal* is stored in a memory within the noise radar equipment, to be read, up-converted, amplified and transmitted. In the radar receiving section a coherent demodulator provides the base-band components (I’, Q’), which are digitally correlated with (I, Q) to form the output of the *correlation receiver* (matched filter) [12]. An equivalent result is often obtained using “low-intermediate frequency” real signals occupying twice the bandwidth, i.e., 2B.

Neglecting at the moment the Doppler mismatching, being both B and T limited, the output of the matched filter, outside of the mainlobe, shows unavoidable random sidelobes, statistically described in [1], whose mean value depends on the integration gain B·T. The presence of these random sidelobes has two damaging effects: first, as said, a strong target may mask a weak one and, second, in continuous emission (CE) operation the coupling between the colocated transmitting and receiving antennas (*leakage* effect) may mask nearby targets, being equivalent to a strong target at nearly *zero* distance. Therefore, the sidelobes of the ACF of the transmitted waveform have to be kept below a given level, defined by a goal sidelobe level below its peak value, i.e., the PSL. For definitions and notations see [1].

Using real hardware, the theoretical performance of the ACF may be corrupted by deleterious effects which increase the PSL as described later.

This paper is organized as follows. Section 2 recalls the waveforms generator for CE-noise radar (CE-NR) introduced in [1], defines the setting of the radar system parameters and discusses the hardware implementation issues related to waveforms. Section 3 and Section 4 describe the noise radar demonstrators which have been implemented at FHR and at TNRCC, respectively, with the related tests and trials in laboratory and in the field. Finally, Section 5 contains discussion of the results, recommendations and conclusions.

## 2. The Noise Waveforms Generator for CE-NR

**Figure 1:**
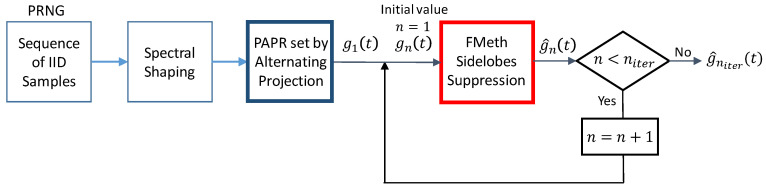
Functional block diagram of the generation of a noise waveform, from [1].

**Figure 2:**
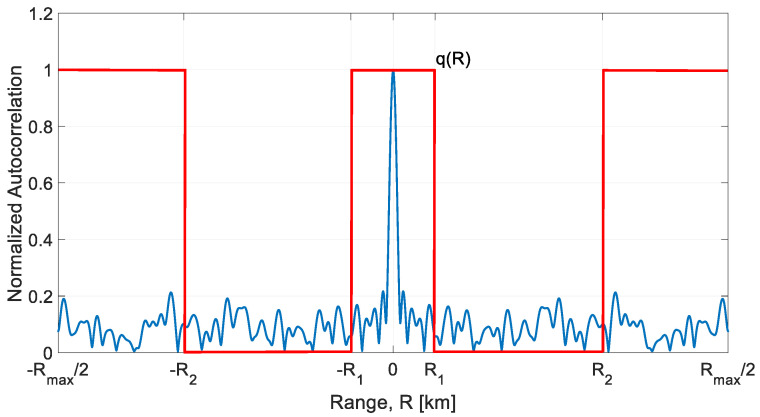
The sidelobes suppression interval $\left(R_{1},R_{2}\right)$ as defined by q(R), red line, from [1].

In [1] an algorithm to generate noise waveforms, with the prescribed bandwidth B, duration T and sampling frequency $F_{s}$, has been proposed and analyzed. Figure 1 recalls the functional block diagram of the algorithm. The aim is twofold: the sidelobe level of the autocorrelation function of the waveform g[k] in the *range* (round trip delay) interval of interest, from $R_{1}$ to $R_{2}$, has to be kept below a prescribed value while the *PAPR* of the waveform has to be set to a design value, e.g., by 1.0, 1.5 or 2.0. The choice of $R_{1}$ and $R_{2}$ (Figure 2) is dictated by the antenna leakage and the nearby clutter level. For more details on radar signals and NR signals, the interested reader is addressed to [13,14,15,16].

The main steps of the generating algorithm are:

(i) ***PRNG and Spectral Shaping***. A sequence of $F_{s}\cdot T$ identically distributed Gaussian complex samples with assigned power spectrum (Spectral Shaping) inside the band B, is generated by a Pseudo Random Numbers Generator (PRNG) and an appropriate filtering [17]. With $F_{s}=B$, the number of independent complex samples (or “degrees of freedom” of the sequence) is BT.
(ii) ***PAPR setting by Alternating Projection.*** After spectral shaping, the PAPR of the sample waveform gets a random “natural value” of 9−12 or greater. To reduce the SNR loss, related to PAPR by $Loss\left[\mathrm{dB}\right]=-10\cdot log_{10}\left(PAPR\right)$, the PAPR is forced to a selected lower value ≥1 (e.g., 1.5−2.0) using the “Alternating Projection” algorithm [18].
(iii) ***FMeth Sidelobes Suppression***. The *FMeth* algorithm allows us to attenuate the PSL in a specific range zone as shown in Figure 2. Due to the folding of the spectral components into (−B/2, +B/2), the output has to be iteratively processed by the *FMeth* algorithm for a selected number of times ($n_{iter}$).

At the end of the procedure, i.e., after $n_{iter}$ iterations needed to satisfy the requirement of the PSL, the “tailored” signal ${\hat{g}}_{n_{iter}}\left(t\right)$ with $t=\frac{k}{F_{s}}$, $k=1,\;2,\ldots ,F_{s}\cdot T$ is used as transmitted waveform.

### 2.1. Parameter Setting in the Waveforms Generator

The input parameters of the waveform generator of Figure 1 are chosen according to:

o Regulations for the usage of the electromagnetic spectrum [6].
o Radar parameters, i.e., sampling frequency $F_{s}$ and intermediate frequency $f_{\mathrm{IF}}$.
o Waveform characteristic (*spectrum shape* and PAPR) as related to the resolution ∆R=c/2B ($c=3\cdot 10^{8}$ m/s), the PSL, the maximum range $R_{max},$ the suppression zone interval $R_{2}-R_{1}$ and the suppression zone attenuation ($n_{iter}$ of the algorithm *FMeth*) including, for a two-antennas CE-NR, the insulation factor between antennas.

An exemplary set of the parameters for a *short-range application* (with an acquisition system based on a modern FPGA board) is shown in Table 1 for the apparatuses, Demonstrator-1 and Demonstrator-2, used for field trials in the marine and land environments.

Two exemplary spectral windows (Hamming and Blackman–Nuttall) were considered among the many available [19,20] to lower the sidelobes of the autocorrelation functions.

**Figure 3:**
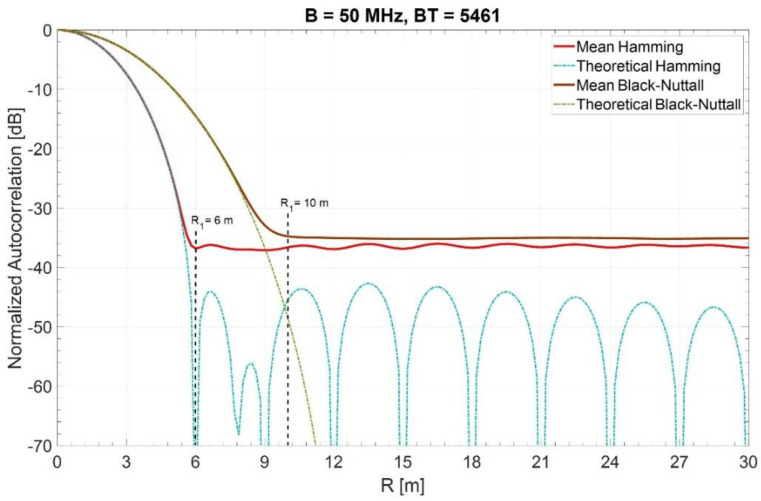
The lower end $R_{1}$ of the suppression zone (mean of 1000 trials) for two spectral windows, Hamming and Blackman–Nuttall [20]. Means estimated by 1000 simulation runs.

For the two spectral windows, Figure 3 shows the mean of the ACF (near the mainlobe) estimated by 1000 simulation runs (with the time converted in range in the abscissa).

The lower end $R_{1}$ is about 6 and 10 m, respectively. For a Blackman–Nuttall noise, outside the mainlobe we observe in Figure 3 a constant plafond (−35 dB) because the theoretical −98 dB of PSL does not influence the mean of the random sidelobes level, whose behavior is analyzed in [1]. Instead, for Hamming noise a slight ripple is visible due to a much greater PSL of about −43 dB and a BT of 5461.

**Figure 4:**
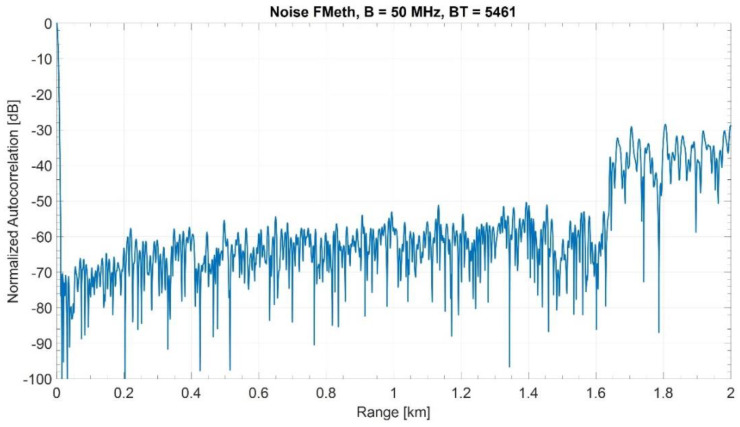
Normalized ACF of a single Blackman–Nuttall noise waveform (PAPR = 9.0) with the 1.638 km suppression zone.

**Figure 5:**
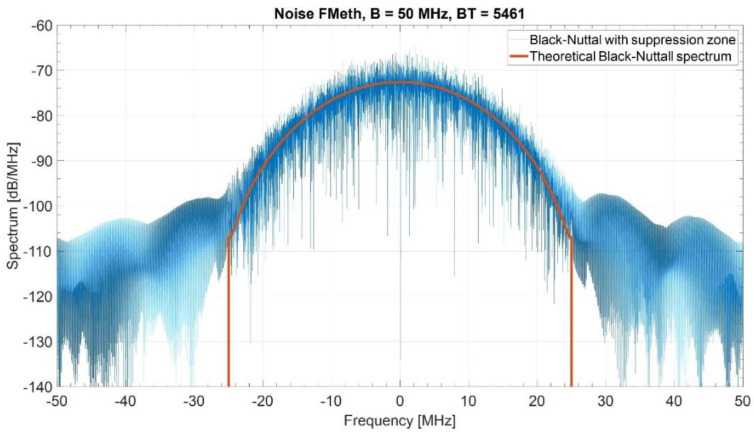
Spectrum of a single Blackman–Nuttall noise waveform (PAPR=9.0).

Figure 4 shows the normalized ACF of a single realization of a Blackman–Nuttall noise waveform with PAPR=9.0 and T=109.22 μs, i.e., a maximum range of 8.19 km. The upper end $R_{2}=\frac{1}{5}R_{max}$ is 1.638 km. In the suppression zone the sidelobe level is below −50 dB. Figure 5 shows the corresponding spectrum as compared with the theoretical one. The attenuation of the sidelobes in the suppression interval causes some widening of the spectrum outside the nominal bandwidth of 50 MHz.

### 2.2. Hardware Implementation Issues

The aim of the experimental investigation, shown in the following, is to illustrate potential damaging effects of hardware imperfections [21] on the expected performance of the radar waveform. Particularly, it is stressed how the quality of the reference signal influences the performance of the radar measurement when tailored waveforms, such as the FMeth class of signals, are used.

Signal quality can be understood in different aspects. Here, we focus on aspects mostly related to the hardware implementation such as quantization and aliasing.

**Figure 6:**
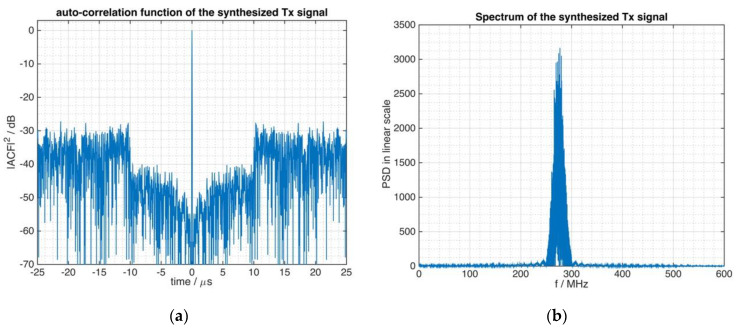
Signal characteristics of the example tailored waveform (FMeth) of B = 50 MHz bandwidth modulated to a carrier frequency of 275 MHz. (**a**) The autocorrelation function, (**b**) the spectrum of the signal.

The exemplary waveform is an FMeth having a PAPR=1.5 and BT=5000 with a Blackman–Nuttal shaped spectrum. This kind of waveform was detailed in [1] and briefly discussed here in Section 2. The experimental investigation uses a modulated representation of the waveform rather than the complex base-band representation that was shown in Section 2. For this reason, Figure 6 displays the autocorrelation function (a) and the resulting spectrum (b) of the test signal and serves as reference for ensuing parts of this section.

#### 2.2.1. Spectral Issues during the Digitization Procedure

The shape of the signal spectrum plays an important role in designing the suppression zone of tailored noise waveforms as detailed in [1]. During the analog-to-digital conversion, aliasing occurs when the conversion violates the well-known Nyquist–Shannon-Kotelnikov criterion [22,23].

The PAPR setting by *Alternating Projection* (Section 2) is a nonlinear processing; hence it modifies the signal’s spectrum by distortion and by creation of harmonics.

The FMeth waveforms, due to their limited PAPR, create frequency components outside of the targeted frequency band. In the examples of this paper, the latter is assumed to be B=50 MHz which is compliant to typical marine navigation radars. In this context, the Nyquist rate is equal to 100 MHz for real-valued representations and 50 MHz for complex valued signals in I, Q representation.

**Figure 7:**
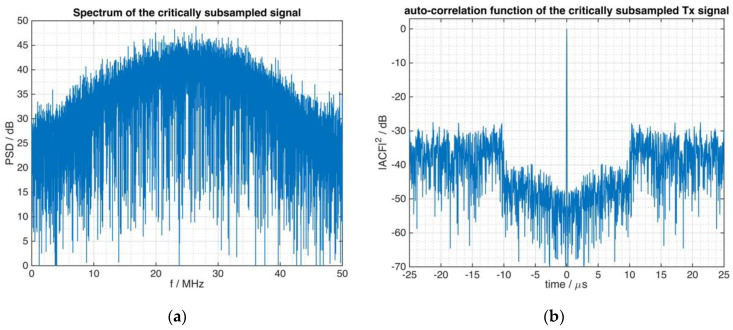
The signal of Figure 6 is subsampled at 50 MHz rate. (**a**) The spectrum is affected by aliasing. (**b**) The autocorrelation function of the subsampled signal.

Figure 7a shows the spectrum of the FMeth signal of Figure 6 from which the digitally subsampled signal was derived. Aliasing effects at the left and right border of the spectrum are observed when Figure 7a is compared to Figure 5.

However, as can be seen from Figure 7b, this spectral issue has little influence on the requested performance concerning the ACF of the signal and the beneficial suppression zone is well preserved, i.e., the ACF is robust against the aliasing signal components. Thus, the critical sampling to the bandwidth of the theoretical Blackman–Nuttall filter function (see Figure 5) that was used during the waveform generation procedure, affects the frequency response and also the autocorrelation function. However, this instance does not result in damaging effects on the suppression zone of the tailored noise signal.

#### 2.2.2. Quantization Effects

Modern components like ADC and digital-to-analog converters (DAC) use a high number of bits (e.g., 16 corresponding to a theoretical dynamic range of 96 dB) and, at a first glance, provide high precision for their output. However, this benefit only applies to full-scale signals, whose amplitude is equal (or close) to the maximum expected input of the ADC and in terms of a DAC, whose digital representation covers the full range of available levels.

**Figure 8:**
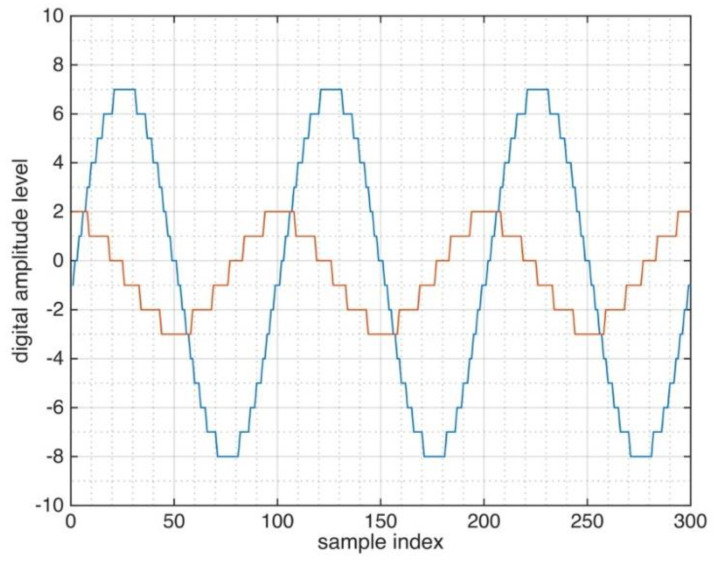
A 4-bit quantization is applied to sine waves of different amplitudes.

In radars, the DAC is used for the digital waveform generation and, thus, the quality of its output signal can easily be assured. Therefore, in the following we focus on the effect of the AD conversion of the reference signal. The general quantization principle is illustrated in Figure 8. Two signals with different amplitude levels are considered and the influence of the amplitude on the effective quantization can be understood from this figure. A 4-bit quantizer is considered that corresponds to integer amplitude levels ranging from −8 to +7. In Figure 8, the full-scale signal (large sine wave) is represented by the full resolution, but the much smaller cosine signal is represented with a much lower resolution by the same quantizer. This phenomenon, in the following, is referred to as the *effective quantization*.

In order to demonstrate how the quality of the radar measurement is influenced by the effective quantization of the replica signal, the synthesized samples of the transmitted signal (Tx) were used and the quantization effect of the ADC was simulated.

**Figure 9:**
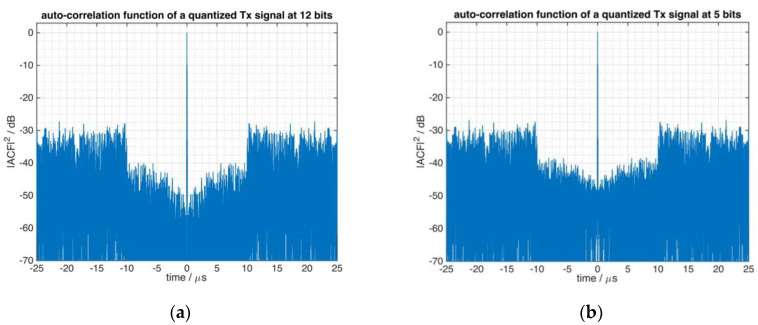

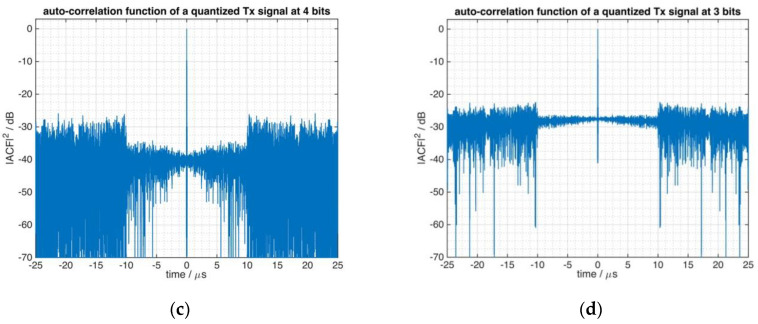
Quantization of tailored waveform at different effective quantization levels: (**a**) 12 bit, (**b**) 5 bit, (**c**) 4 bit, (**d**) 3 bit.

The FMeth waveform of Figure 6 was quantized as a full-scale signal by four different ADCs with resolutions of 12, 5, 4 and 3 bit, and the autocorrelation functions of the quantized signals were calculated in order to rate the effect of the quantizing step. The set of autocorrelation functions is shown in Figure 9 which highlights that the higher the effective quantization of the ADC, the closer it approximates the ideal shape of the ACF (as was drawn in Figure 6) of the tailored radar waveform.

When the effective quantization level is of the order of 4 bit or less, the ACF of the signal is severely affected concerning the sidelobe suppression zone. Consequently, for tailored waveforms it is important to have a good enough degree of quantization of the replica signal in order to allow for a correct implementation of these waveforms in a given application. Figure 9 shows the quantization effect of the reference signal with four different levels, a variation mostly due to the signal power.

The reason for the observed damages to the suppression zone of the tailored noise signal corresponds to the waveform creation. As was discussed in [1], the quality of the amplitude representation (there, in terms of the PAPR) influences the number of degrees of freedom that the waveform generation algorithm utilizes in order to achieve the desired signal performance. If, on the other hand, a low effective quantization quality later removes amplitude information from the reference signal, a vital amount of information contained in the waveform (that was required to create the suppression zone in the autocorrelation function) seems to get lost.

**Figure 10:**
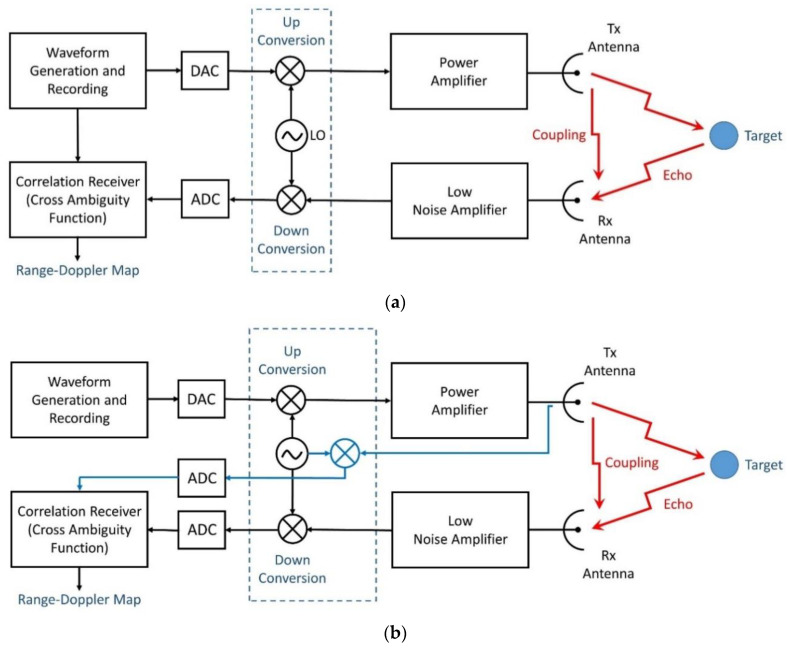
Basic block diagram, from [1]. (**a**) The reference is the digital record of the transmitted code; (**b**) the reference is the record of the transmitted signal at the antenna port; ADC = analog-to-digital converter; DAC = digital-to-analog converter.

Summing up, when the reference for the correlation receiver (Figure 10b) is taken from the transmitted signal, care must be taken concerning the transmitted power, as for low power levels the quantization effects may significantly affect the output of the correlation receiver.

## 3. Noise Radar Trials at Fraunhofer FHR

### 3.1. Experimental Setup

The FHR-noise radar demonstrator (FHR-NR), designed and built at Fraunhofer FHR, is of the CE type and uses two staring horn antennas, with a sheet of isolating material between both. One antenna is used for transmission and the other one for reception. In order to allow for many different scientific investigations, the radar processing is performed offline. The raw radar signals are recorded at a sampling frequency of 250 MHz and represent the surveillance echoes. Furthermore, a replica of the transmitted waveform, also sampled at 250 MHz, serves as the reference signal of a matched filter in the radar processing.

The waveform generator of the X-band transmitter operates at the much higher sampling rate of 1250 MHz. The integer fraction of the transmitter sampling frequency to the receiver sampling frequency allows for the usage of both, analog and synthetic, reference signals in the offline radar processing, see Table 1. Thus, this setup covers both design approaches, that were introduced in [1], i.e., the radar’s replica (reference signal) can either be transferred from the transmitter to the receiver digitally (Figure 10a) or it can be acquired by the receiver from an analog copy of the transmitted signal (Figure 10b). Both options are used in the ensuing analysis.

**Figure 11:**
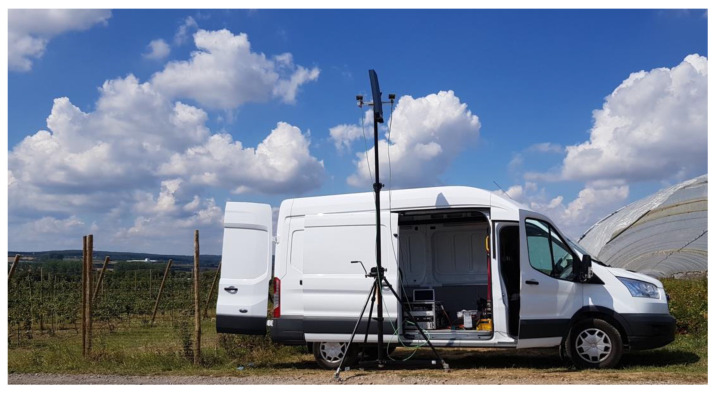
The FHR-NR demonstrator allows for a flexible setup.

In the framework of this paper, the FHR-NR demonstrator (Figure 11) is used to investigate the relationship of the quality of the reference signal on the performance of the radar measurement.

**Figure 12:**
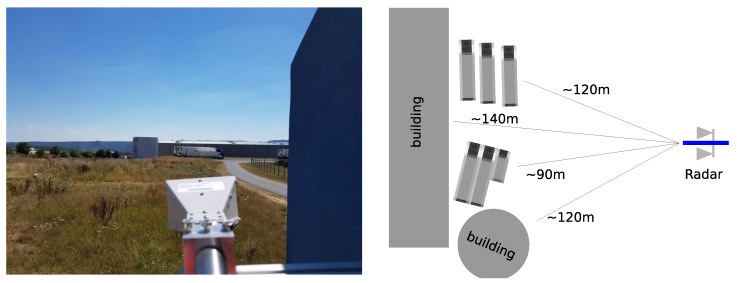
The recorded scene contains two large storage buildings and a set of parked trucks. The ranges to the different targets are similar and the wide beam of the radar antenna allows for their simultaneous illumination.

It was expected that the complex arrangement of large radar targets, as shown in Figure 12, benefits from the usage of tailored noise radar waveforms.

### 3.2. Experimental Results

Neither are quantization nor spectral folding expected to occur on the transmitter side of this radar as the DAC is fed by a full-scale signal and operates at a sampling frequency much higher than the Nyquist rate pertaining to the waveform to be transmitted. However, when the power level of the replica signal is too low, the tailored waveform used in this experiment is not fully exploitable.

**Figure 13:**
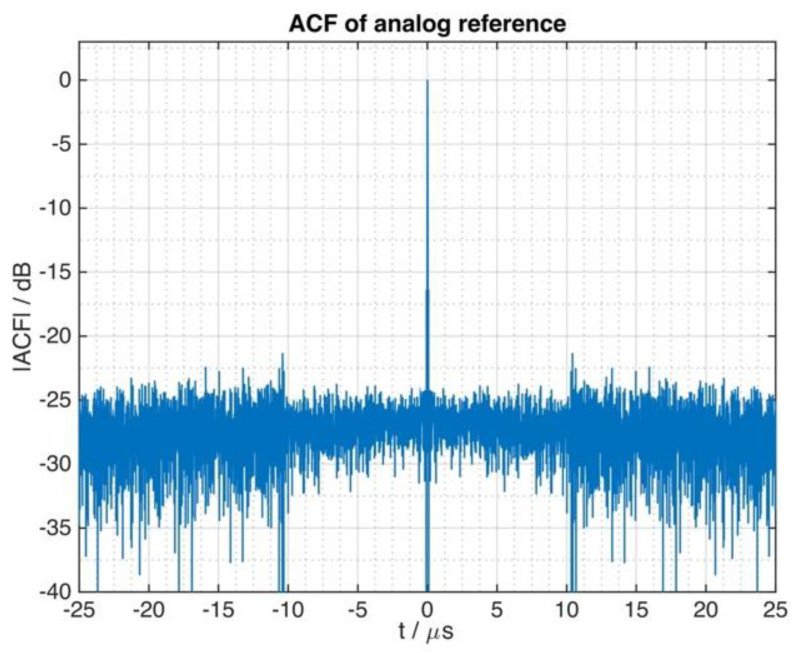
The autocorrelation function of the digitized reference signal differs significantly from its ideal shape (please refer to Figure 6). Instead it shows similar impairments as those signals of Figure 9 that were affected by the quantization.

The autocorrelation function of the analog reference signal, recorded during the experiment, indicates the main reason of this issue. Its shape (see Figure 13) is very similar to the shape of the autocorrelation functions as shown in Figure 9, which illustrated the influence of the quantizer on the radar performance.

**Figure 14:**
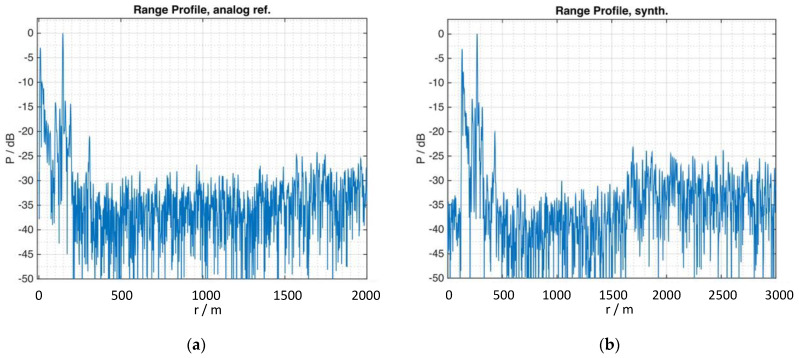
The same set of data shows different qualities when processed with the analogue reference (**a**) or with the synthetic reference signal (**b**).

This issue has a dramatic effect in the performance of the real-world radar measurement of this section. For illustration purposes, the same set of data of a range measurement was processed with two different replica (reference) signals. First, shown in Figure 14a, the reference signal is obtained from the analog front-end of the radar (according to Figure 10b), second, shown in Figure 14b, a subsampled copy of the synthetic waveform of Figure 6 (according to Figure 10a) was used for the matched filter process. The difference in quality can be clearly identified from this display.

From Figure 14a we obtain the following information: first target (close-in clutter and leakage) at 9.6 m, strongest at 148 m, far-most at 308 m; the strongest is accompanied with two spikes at 102 and 193 m.

These results indicate two important conclusions. First, the quality of the reference signal in radars that use tailored noise waveforms needs to be taken care of as the two extreme situations of the simulations that were made in Section 2.2.2 were verified experimentally on the same set of data by using two differently quantized replicas of the transmitted waveform. Second, Figure 14a shows that even if important signal information on the suppression zone, the most important feature of tailored noise signals, get lost, the resulting signal still remains, what it basically is, a pseudo-random and non-repetitive signal. Thus, the performance of an ordinary noise radar can be seen as the lower boundary of the performance of a noise radar with tailored waveforms.

## 4. Noise Radar Trials at TNRCC

### 4.1. Noise Radar Demonstrator

The experimental noise radar demonstrator designed and developed at TNRCC has similar performance to that of a typical low-power short range marine radar. Operating in X-band, the demonstrator was built with the goal of investigating noise radar technology and noise radar waveforms in the maritime domain. It employs continuous emission and thus has two colocated microstrip antennas for transmission and reception with isolation baffles in between to provide leakage isolation. The architecture of the radar conforms to Figure 10b where a replica of the transmit signal is obtained in the analog reference channel and thereafter digitized by the analog-to-digital converter for matched filtering.

The demonstrator has an FPGA-based backend for driving the transmitter with noise waveforms and matched filter processing of digital surveillance and reference signals. The FPGA hosts an internal PRNG capable of generating noise sequences in real time and a waveform buffer for synthetic (tailored) waveforms generated *a priori*. The operator can choose either PRNG for nonrepetitive noise waveform transmission or waveform buffer for repetitive tailored waveform transmission. Although configurable, the DAC and ADC sampling rates were set to 1200 and 200 MSPS, respectively, for the trials reported in this paper.

The demonstrator has two modes “*operation”* and “*engineering*”*,* where in the former the processed and low-throughput (down-sampled) radar data is produced by the radar and sent to a PC for real time display and in the latter, raw and high-throughput data is produced by the radar and sent to a PC for recording into storage.

In this particular trial, the demonstrator was used in *engineering* mode, in order to capture raw reference and surveillance channel signals for offline processing on the PC.

In the context of this paper, the demonstrator is used to investigate the practical problems encountered in a typical noise radar operation. The effects of close strong targets and near-in ground clutter were analyzed and the dynamic range improvement is shown with the usage of tailored noise radar waveforms.

### 4.2. Description and Analysis of the Test/Trials Results

The trials were conducted in a shore-based test facility in Istanbul, Turkey. In the trials campaign, FMeth waveform was tested against trihedral corner reflector and a fishing boat (target of opportunity). The results were compared with those obtained using Gaussian noise waveform (generated by the Internal PRNG).

In the following sections, the effect of a close strong scatterer and near-in clutter on the dynamic range was analyzed and a surveillance scan (PPI—Plan Position Indicator-view) is presented to show how effectively FMeth waveforms can be used for the suppression of sidelobes in the range of interest.

#### 4.2.1. Corner Reflector at 130 m (Staring Mode)

**Figure 15:**
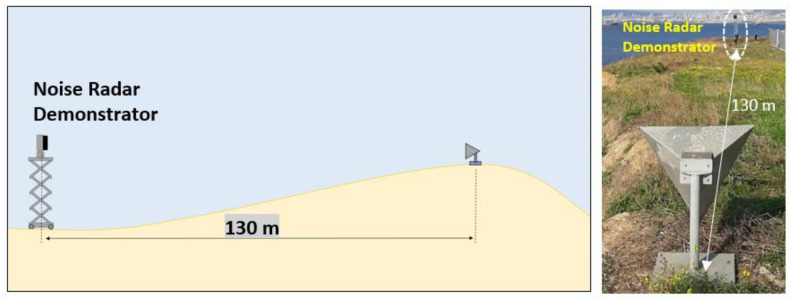
Corner reflector measurements scenario using Demonstrator-1.

In this scenario, a trihedral corner reflector having a reflectivity of 27 dBsm (in X-band) was placed 130 m away from the radar. As shown in Figure 15, there is a slope towards the corner reflector and beyond the ramp where the corner reflector was located; hence, some echoes are expected due to geographical limits obscuring the sight of radar. This allowed us to better analyze and visualize the sidelobe behavior of FMeth waveform for the ranges beyond the corner reflector.

**Figure 16:**
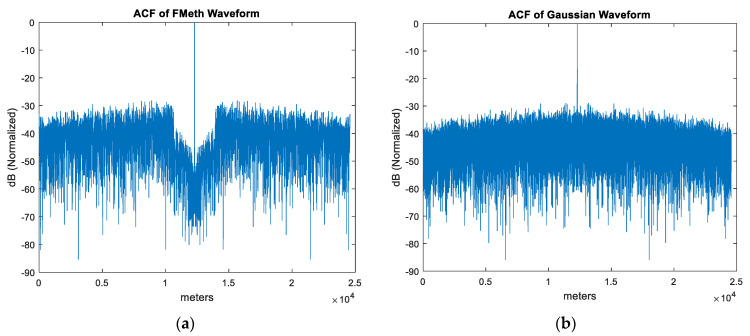
Autocorrelation function of waveforms: (**a**) FMeth waveform, (**b**) Gaussian noise (PRNG).

During the trials, raw data was recorded for the FMeth waveforms and Gaussian noise waveforms (Internal PRNG). The ACF of FMeth and Gaussian noise waveforms are shown in Figure 16. As expected, the Gaussian noise waveform provides a BT of 5461 (50 MHz bandwidth and 109.22 μs duration) leading to a sidelobe level of 37.4 dB. On the other hand, the ACF of the FMeth waveform exhibits lower sidelobes inside the suppression zone than Gaussian noise. However, it should be noted that due to fixed point quantization of signals (dictated by ADC), the FMeth sidelobes inside the suppression zone are ~10 dB higher as compared to ACF plot shown in Figure 4 which is calculated on PC using floating point precision.

**Figure 17:**
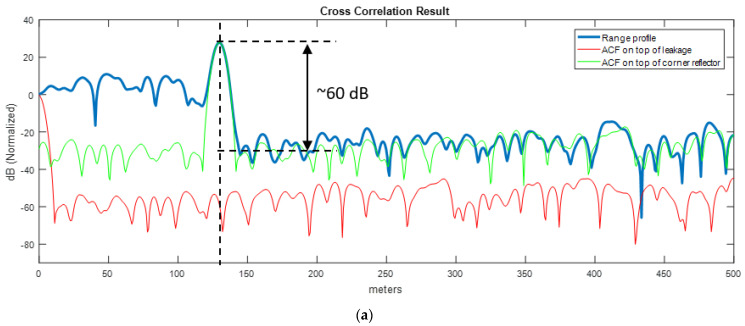

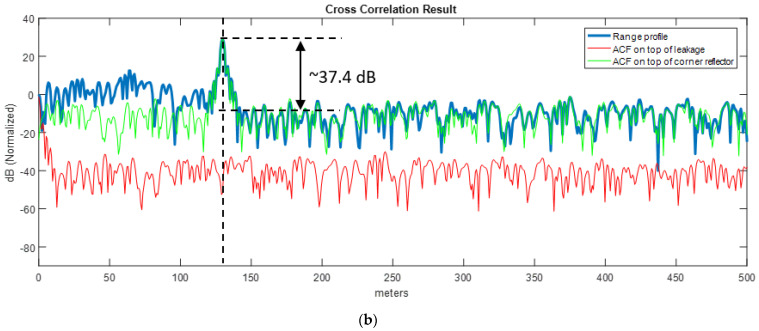
Range profiles with corner reflector at 130 m. (**a**) FMeth waveform, (**b**) Gaussian noise (PRNG).

In Figure 17, the range profiles up to 500 m are shown for both waveforms. The corner reflector is clearly seen in both plots at 130 m. The ACF of each corresponding waveform is put on top of the leakage at zero distance and on top of the target at 130 m to show the sidelobe behavior of both waveforms. As mentioned earlier, the sight of radar is obscured by the hill and no echo was captured at range beyond the target. By simple observation on range profiles, we can draw the conclusion that the range swath beyond the corner reflector is dominated by the sidelobes of the corner reflector for both waveforms. The actual range profile (blue line) is in good agreement with the sidelobes of waveforms (green line) on the righthand side of peak the response of the corner reflector.

This trial shows us that a strong scatterer near the radar as given in the scenario can easily desensitize the radar and cover the whole range profile with its sidelobes. Hence, during the waveform design stage, sidelobes should be lowered to improve the matched filter dynamic range. For example, in FMeth case (Figure 17a), the sidelobe level is about −60 dB below the peak response whereas it is around −37.4 dB in the Gaussian noise waveform case (Figure 17b). In this respect, having reduced sidelobes using tailored waveforms may mitigate dynamic range problems in operational noise radar applications.

#### 4.2.2. Fishing Boat (Staring Mode)

**Figure 18:**
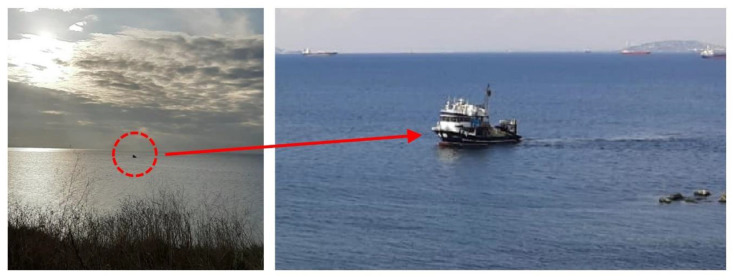
Fishing boat at 800 m from the radar.

In this trial, a moving fishing boat shown in Figure 18 was spotted within the 1.5 km range. The radar being in staring mode, the raw radar signals were recorded for both FMeth and Gaussian noise waveform. Aside from the effects of strong scatterer shown with corner reflector trials in the previous section, here we focus on the near-in ground clutter effects which may raise the dynamic range floor of the matched filter output.

**Figure 19:**
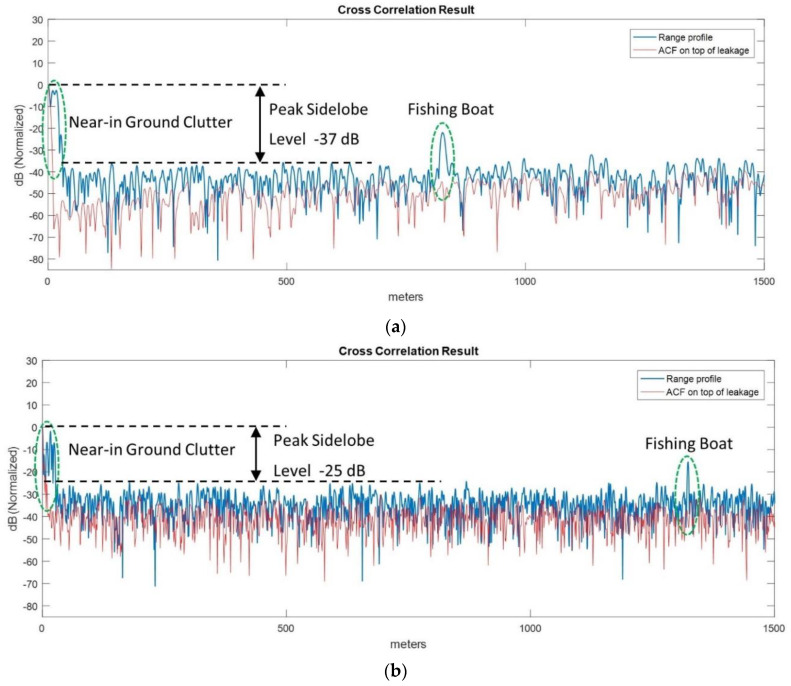
Range profiles with fishing boat: (**a**) FMeth waveform, (**b**) Gaussian noise (PRNG).

In Figure 19 we show the two range profiles obtained using FMeth and Gaussian noise waveforms. Due to the time delay between the measurements, the boat moved 522 m away from its initial position.

An important aspect of this trial is the effect of near-in ground clutter on the dynamic range. In Figure 19 the red line denotes the ACF of leakage response at *zero* distance and we would consider its sidelobes as the dynamic range floor of the matched filter output if there was no clutter nearby.

Due to the near-in ground reflections (clutter), the sidelobes of clutter add up and raise the matched filter output floor as shown in the actual range profile (blue line). Therefore, the near-in ground clutter can be deleterious in this regard. Any objects to be detected must be observable well above (e.g., +10 dB) the dynamic range floor (black dashed line) dominated by the near-in clutter sidelobes.

As seen in Figure 19a, FMeth grants lower sidelobes (i.e., ~−37 dB) than Gaussian noise waveform counterpart (i.e., ~−25 dB) which leads to better target SNR for the ensuing plot extraction and target tracking.

#### 4.2.3. Marine Surveillance Trials (Scanning Mode)

In typical marine navigation, the ship operators may be more interested to observe the near range zone for incoming vessels and low visible objects on sea surface for safety concerns. In this regard, setting a radar suppression zone as shown in FMeth procedure for the radar might aid in this navigational need. In this trial, we showed how effectively the suppression of sidelobes is achieved by the FMeth waveform for the desired range of interest.

For the purpose of demonstration, the radar was operated in scan mode with a 15 RPM rotational speed. The waveform Repetition Frequency was set to 1 kHz and a single scan image was recorded.

**Figure 20:**
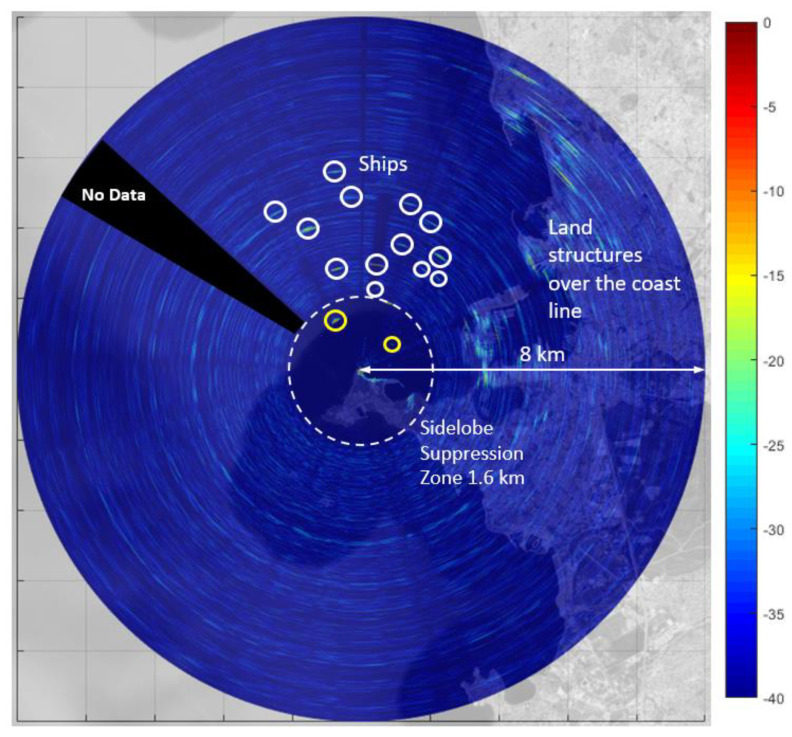
PPI scan image obtained using FMeth waveform. The suppression zone is 1.6 km (dashed white circle). Ships are spotted in white and yellow circles.

In Figure 20 a Plan Position Indicator (PPI) view of the scene is shown. The PPI image was constructed by overlaying the recorded data on a geographical map. The black shaded area shows a *dead* zone where data was not captured due to data overflow.

In the PPI image, the spotted ships are encircled in white. Land structures such as buildings are visible over the coast line. The large dashed circle shows the boundary of suppression region (~1.6 km) where the dynamic range floor is clearly well below −40 dB according to the color-bar. Two targets (marked with yellow circles) inside the suppression zone are quite visible due to low sidelobes around the target response. It is expected that plot extraction and target tracking performance would be better (high SNR) inside the sidelobe suppression zone as compared to the out of suppression zone, where the sidelobes may raise the dynamic range floor, resulting in low SNR.

## 5. Lessons Learnt—Comments, Recommendations and Conclusions

The theory of noise waveforms and their generation described in the companion paper [1], was implemented to carry out experimental results using two different X-band noise radar demonstrators, both of continuous emission type, at the FHR Fraunhofer Institute in Wachtberg (Germany) and at the TNRCC in Istanbul (Turkey). As this paper is focused on generation and processing of noise radar waveforms rather than on specific equipment, a detailed description of the hardware is omitted.

As a general result, the architecture of low-power, continuous emission noise radar was tested in different environments showing its suitability for short-range, high-resolution surveillance.

In both land and marine environments, targets were detected in the presence of clutter and of antenna leakage. In the marine environment, in spite of the very low transmitted power, a Plan-Position-Indicator (PPI) was generated showing opportunity targets up to five kilometers.

The experiments confirmed the need for tailoring the transmitted waveform to improve the dynamic range of the matched filter output and to counteract the damaging effects of the leakage and of the nearby clutter and strong targets.

In the receiving section, a continuous emission noise radar digitally correlates the transmitted signal (reference signal) with the received one. A first analysis has concerned the effect of the quantization on the reference signal. As described in [1], two architectures were considered. First, the reference signal was directly digital recorded from the transmitted code. Second, the reference signal was recorded at the antenna port. The comparison of both architectures by the same set of data revealed the requirement of tailored noise waveforms for a high quality of the reference signal. However, the results of this paper indicate that even if “things go wrong”, the remaining performance of the tailored noise waveforms is not worse than the performance of conventional noise signals. This fact can be considered as fallback solution or safety net when dealing with tailored noise signals.

## Figures and Tables

**Table 1:** Parameters for short-range applications.

Parameters	Demonstrator-1	Demonstrator-2
Band, B	50 MHz	50 MHz
Sampling frequency, $F_{s}$	1200 MSPS (24·B)	1250 MSPS (25·B)
Number of samples, $n_{s}$	$2^{17}=131,072$	125,000
Duration, $T=\frac{n_{s}}{F_{s}}$	109.22 μs	100 μs
Integration gain, BT	5461 (37.4 dB)	5000 (37.0 dB)
Peak-to-average power ratio, PAPR: variable	1.0−9.0	1.0−9.0
Maximum range, $R_{max}$	8.19 km	7.50 km
Sidelobes suppression zone, $R_{2}$ $\left(R_{1}≅0\right)$	$\frac{R_{max}}{5}$	$\frac{R_{max}}{5}$
Number of iterations of FMeth, $n_{iter}$	100	100
